# Candidate genes for obstructive sleep apnea in non-syndromic children with craniofacial dysmorphisms – a narrative review

**DOI:** 10.3389/fped.2023.1117493

**Published:** 2023-06-27

**Authors:** Zuzana Marincak Vrankova, Jan Krivanek, Zdenek Danek, Jiri Zelinka, Alena Brysova, Lydie Izakovicova Holla, James K. Hartsfield, Petra Borilova Linhartova

**Affiliations:** ^1^ Clinic of Stomatology, Institution Shared with St. Anne's University Hospital, Faculty of Medicine, Masaryk University, Brno, Czech Republic; ^2^ Clinic of Maxillofacial Surgery, Institution Shared with the University Hospital Brno, Faculty of Medicine, Masaryk University, Brno, Czech Republic; ^3^ RECETOX, Faculty of Science, Masaryk University, Kotlarska 2, Brno, Czech Republic; ^4^ Department of Histology and Embryology, Faculty of Medicine, Masaryk University, Brno, Czech Republic; ^5^ E. Preston Hicks Professor of Orthodontics and Oral Health Research, University of Kentucky Center for the Biologic Basis of Oral/Systemic Diseases, Hereditary Genetics/Genomics Core, Lexington, KE, United States

**Keywords:** pediatric obstructive sleep apnea, syndrome, craniofacial dysmorphism, candidate gene, skeletal anomaly

## Abstract

Pediatric obstructive sleep apnea (POSA) is a complex disease with multifactorial etiopathogenesis. The presence of craniofacial dysmorphisms influencing the patency of the upper airway is considered a risk factor for POSA development. The craniofacial features associated with sleep-related breathing disorders (SRBD) – craniosynostosis, retrognathia and micrognathia, midface and maxillary hypoplasia – have high heritability and, in a less severe form, could be also found in non-syndromic children suffering from POSA. As genetic factors play a role in both POSA and craniofacial dysmorphisms, we hypothesize that some genes associated with specific craniofacial features that are involved in the development of the orofacial area may be also considered candidate genes for POSA. The genetic background of POSA in children is less explored than in adults; so far, only one genome-wide association study for POSA has been conducted; however, children with craniofacial disorders were excluded from that study. In this narrative review, we discuss syndromes that are commonly associated with severe craniofacial dysmorphisms and a high prevalence of sleep-related breathing disorders (SRBD), including POSA. We also summarized information about their genetic background and based on this, proposed 30 candidate genes for POSA affecting craniofacial development that may play a role in children with syndromes, and identified seven of these genes that were previously associated with craniofacial features risky for POSA development in non-syndromic children. The evidence-based approach supports the proposition that variants of these candidate genes could lead to POSA phenotype even in these children, and, thus, should be considered in future research in the general pediatric population.

## 1. Introduction

Both pediatric (POSA) and adult obstructive sleep apnea (OSA) count among sleep-related breathing disorders (SRBD). POSA is considered a multifactorial disease triggered by the combination of genetic predispositions and several risk factors, including obesity, neuromuscular factors, adenotonsillar hypertrophy, and specific craniofacial features (1, 2). In adults, the genetic background leading to the OSA phenotype has been studied more intensively than in children.

So far, several studies on candidate genes, phenome-wide association studies of OSA genomic variation, and genome/phenome-wide association studies (GWAS/PheWAS) on adult patients with OSA have been published (3–5), while only a single GWAS focusing on children has been reported (6). That study included 1,486 subjects, 1 week to 18 years old, 46.3% of whom were European-Americans and 53.7% African-Americans. The study identified genomic loci associated with POSA at 1p36.22, 15q26.1, 18p11.32 (rs114124196), 1q43 (rs12754698), 2p25 (rs72775219). 8q21.11 (rs6472959), 11q24.3 (rs4370952), and 15q21.1 (rs149936782); children with craniofacial disorders were excluded from that study (6).

Moreover, single nucleotide polymorphisms (SNPs) in genes encoding apolipoprotein E, fatty-acid binding protein 4, nicotinamide adenine dinucleotide phosphate (NADPH) oxidase, and the macrophage migration inhibitory factor were associated with increased or decreased odds of POSA development in children (7–9). These genes are considered to be candidate genes for POSA development (i.e., they are likely to be related to this disease because of their genomic location or known function). All four mentioned genes are associated with lipid metabolism and/or immune system function. It is, therefore, possible that the susceptibility of carriers of these SNPs to POSA is associated with their role in the development of obesity.

However, genetic background is involved, to some extent, in all of the most commonly reported POSA risk factors – besides obesity, body fat distribution, ventilation control mechanisms, upper airway neural control, and soft tissue morphology, genetic background plays a role also in craniofacial dysmorphisms (10–13). In this narrative review, we closely focus on specific genes involved in the development of the orofacial area and of certain craniofacial features, which makes them possible candidate genes for POSA. Thus, we aimed to (i) describe craniofacial anomalies associated with POSA development, (ii) select syndromes characterized by severe craniofacial dysmorphisms associated with OSA and/or high prevalence of pediatric SRBD, (iii) summarize information about the genetic background of these syndromes, and (iv) suggest candidate genes for POSA in non-syndromic patients with craniofacial dysmorphisms.

## 2. Craniofacial characteristics associated with POSA development

As the upper airway dimensions and morphology of the craniofacial area are closely related, it is no surprise that some abnormalities in its soft and bony structures may contribute to the narrowing and easier collapse of the airway, resulting in OSA, both in children and adults (14–16). Patients suffering from severe skeletal craniofacial malformations could be at a three times higher risk of POSA development than the general pediatric population (17). The importance of craniofacial morphology in OSA development was confirmed also by Kim et al., who reported the presence of craniofacial dysmorphisms, such as the narrow nasomaxillary complex or underdeveloped mandible, in 93.3% of children diagnosed with sleep-disordered breathing (18).

**Figure 1:**
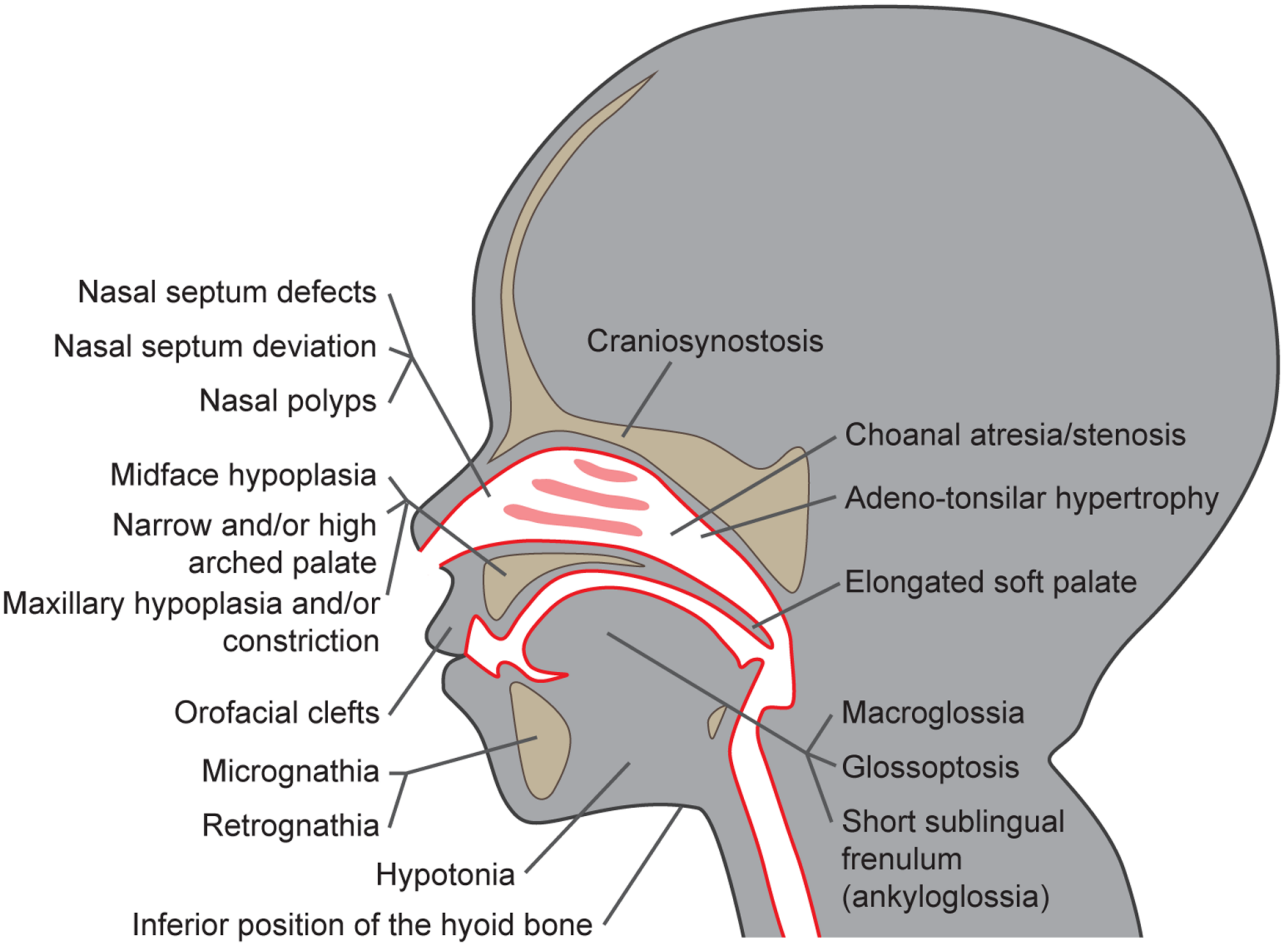
Craniofacial dysmorphisms as risk factors for pediatric obstructive sleep apnea development.

Multiple studies described craniofacial characteristics that are more often present in children suffering from SRBD than in children without these conditions (19–24). These include the size of the maxillo-mandibular complex, their (absolute and mutual) position, and growth pattern, as well as dental occlusion and facial appearance. The craniofacial dysmorphisms associated with the increased risk of POSA development are summarized in Figure 1. Extended facial profile and retrognathia have also been suggested to be more common in children with OSA; however, a recent systematic review by Fagundes et al. did not confirm this association (25).

### 2.1. Skeletal anomalies in the orofacial area risky for POSA development

Premature bone fusion, craniosynostosis, is one of the key features playing role in the narrowing and easier collapse of the airways. It is often diagnosed together with midface hypoplasia, i.e., a combination of the underdevelopment of the maxilla, cheekbones, and eye sockets (although both these features may occur also independently). Even though these features are well-recognized factors in POSA development, the etiology is usually multifactorial and many children suffer from multilevel airway obstruction (24). Underdevelopment of the upper jaw, i.e., maxillary hypoplasia or, in the case of more pronounced narrowing, maxillary constriction, which are often associated also with narrow and/or high arched palate and lateral crossbite, are other characteristics often present in children suffering from POSA (19, 23, 24, 26). Severe reduction of the naso- and oro-pharyngeal airway space may be present in children with craniosynostosis, in patients with clefts originating from prenatal incomplete tissue fusion (20, 21, 27), or in those with anomalies of the mandible, especially if the mandible is undersized, (i.e., micrognathia; (19, 23, 24, 26). However, the underdevelopment of the maxillo-mandibular complex is not the only factor decreasing the airway patency. According to cephalometric studies, sagittal and vertical maxillo-mandibular complex discrepancies, such as mandibular retrognathia, often diagnosed as skeletal class II malocclusion, and increased overjet or open bite, which may appear due to increased mandibular plane angle, are overrepresented in children diagnosed with POSA (22, 26, 28, 29). This hyperdivergent skeletal pattern may lead to the development of the long-face syndrome, which is another facial appearance typical of patients with SRBD (28, 30, 31). Negative anterior overjet and skeletal class III malocclusion are not as often associated with POSA; still, the association is possible, especially if they are caused by severe maxillary deficiency (32). The lower position of the hyoid bone is another skeletal risk factor that can be diagnosed in a cephalogram. As some lingual muscles insert on that bone, their pull in a downward direction can also cause the narrowing of the airway space and, in effect, apnea (14, 33–35).

### 2.2. Soft tissue anomalies in the orofacial area risky for POSA development

The morphology of soft tissues plays an important role, too. Adeno-tonsillar hypertrophy is a well-described etiological factor of POSA. The deviation or deformity of the nasal septum, hypertrophy of nasal turbinates, or nasal polyps may also increase nasal resistance (36–38) and contribute towards mouth breathing, often accompanied by unphysiological head posture, insufficient lip seal or open bite, all of which are characteristics often present in patients with POSA (16). The lack of nasal breathing accompanied by an imbalance in muscle activity, often associated with the hypotony of orofacial muscles, have a huge impact on the development and growth of the maxillo-mandibular complex and may contribute to its abnormal shape and size (16, 39, 40).

The tongue is another factor playing a key role in the narrowing and collapse of the upper airway. The short sublingual frenulum (or ankyloglossia) in its most severe form leads to a low tongue position and disrupted tongue movement and has been already associated with the POSA phenotype (16, 41–43). An insufficient stimulation of the palatal suture, caused by this unphysiological tongue position, may result in the formation of a narrow palate and decreased volume of nasal cavities, which, again, contributes to the preference for mouth breathing and airway narrowing (16, 33). POSA has also a high prevalence in patients with glossoptosis, which is a down- and backward position of the base of the tongue (44). The combination of glossoptosis with micrognathia or retrognathia leads to a high risk of tongue-based airway obstruction (24, 45). In addition, macroglossia and/or an elongated soft palate could reduce airway volume and contribute to airway obstruction (14, 34).

These craniofacial characteristics are associated with several syndromes; however, they could be also found in non-syndromic children (14, 15, 19). Even though they are usually present in less severe forms, they could still contribute to airway obstruction. Craniofacial features associated with POSA could be easily diagnosed and their heritability is estimated to be high. This is especially true for the size of the maxillo-mandibular complex and the timing of its growth (11, 13, 46).

## 3. Craniofacial syndromes associated with a high prevalence of pediatric SRBD

The prevalence of SRBD, including POSA, may be very high in syndromic children with a severe form of craniofacial dysmorphism. In a population-based case-control study, an OSA diagnosis was associated with the presence of craniofacial anomalies, in particular with orofacial clefting and Down syndrome (46). To better understand the role of genetic factors in both POSA and craniofacial anomalies associated with this diagnosis, we have reviewed the current body of literature and selected syndromes, which: (1) are characterized by severe craniofacial abnormalities associated with POSA, (2) have a high prevalence, or have been already related to the co-incidence of SRBD and POSA in children, and (3) have a known genetic background.

Based on these criteria, 26 syndromes and disorders were selected, namely achondroplasia, Antley-Bixler, Apert, Auriculocondylar, Beare-Stevenson, Cohen, and Collins syndromes, congenital central hypoventilation, craniofacial microsomia (Goldenhar syndrome, oculo-auriculo-vertebral spectrum), craniofrontonasal dysplasia, Crouzon, Down, Ehlers-Danlos, Ellis-van Creveld, Jackson-Weiss, Marfan, and Marshall-Stickler syndromes, mucopolysaccharidosis IV and VI, Muenke, Noonan, abd Pfeiffer syndromes, Pierre Robin sequence, Prader-Willi, Saethre-Chotzen and Treacher-Collins syndromes. From the craniofacial dysmorphisms associated with OSA, craniosynostosis, oral clefts, midface and maxillary hypoplasia, narrow high-arched palate, micrognathia, retrognathia, choanal atresia, macroglossia, and glossoptosis were the features found most frequently in these syndromes (21, 4–70). It is necessary to mention that in syndromes associated with high POSA prevalence, a combination of several of these features is often present. For example, the Pierre Robin sequence associated with high POSA prevalence consists of the following: micrognathia, glossoptosis, narrow and/or high-arched palate, and cleft palate (45).

The information about the genetic background and prevalence of SRBD in these syndromes, including POSA, is summarized in Supplementary Table S1 in the Supplement. The prevalence of pediatric SRBD in children suffering from the mentioned syndromes ranges between 10%–87.5%, which is much higher than in the common pediatric population (2%–4%) (21, 45, 53, 54, 56, 58, 63, 69–87). High prevalences of SRBD were found particularly in populations of children with Treacher-Collins syndrome, mucopolysaccharidosis IV and VI, Apert, and Prader-Willi syndrome, in which limited midfacial development is a characteristic feature (21, 53, 54, 69, 70, 72). Despite their shared relationship to craniofacial dysmorphisms and high SRBD prevalence, these syndromic phenotypes are associated with different genes. In total, aneuploidy in Down syndrome and variations in 30 genes in the other 25 mentioned syndromes (see Supplementary Table S1 in the Supplement) are considered causative or risk factors for SRBD development.

## 4. Possible candidate genes for POSA development in children with craniofacial dysmorphisms

**Figure 2:**
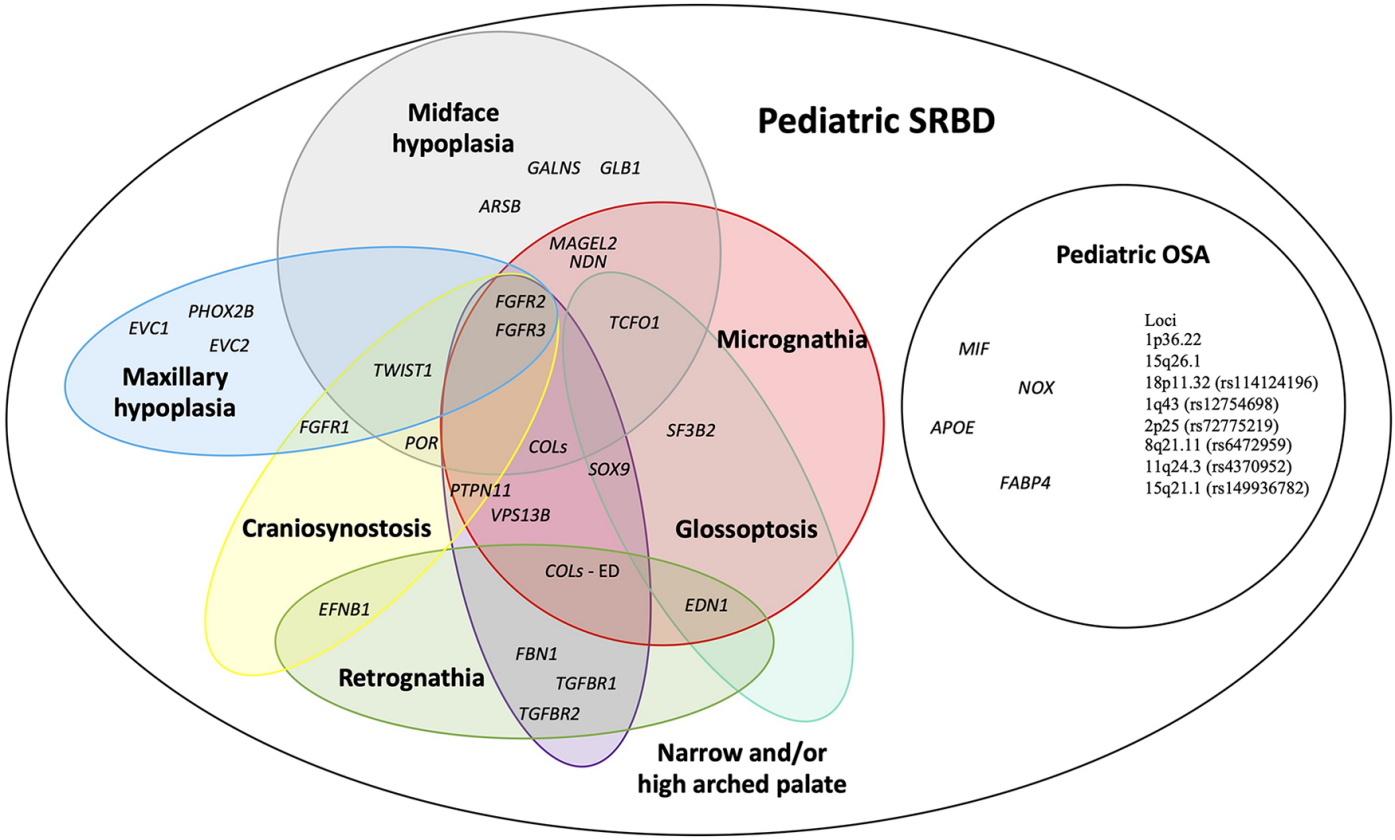
An overview of possible candidate genes for pediatric sleep-related breathing disorders (SRBD), especially pediatric obstructive sleep apnea (POSA) in children with craniofacial dysmorphisms. *APOE*, apolipoprotein E; *ARSB*, N-acetylgalactosamine-4 sulfatase; *COLs* (- ED), collagens gene family (genes associated with Ehlers-Danlos syndrome); *EFNB1*, ephrin-B1*; EDN1*, endothelin 1; *EVC1,* EvC ciliary complex subunit 1; *EVC2,* EvC ciliary complex subunit 2*; FABP4,* fatty acid-binding protein 4; *FBN1*, fibrillin 1; *FGFR1*, fibroblast growth factor receptor 1; *FGFR2*, fibroblast growth factor receptor 2; *FGFR3*, fibroblast growth factor receptor 3; *GALNS,* galactosamine-6-sulfatase; *GLB1*, b-D-galactosidase; *MAGEL2*, MAGE-like protein 2; *MIF*, macrophage migration inhibitory factor; *NDN*, necidin; *NOX,* NADPH oxidase 1; *PHOX2B*, paired like homeobox 2B; POR, cytochrome P450 oxidoreductase; *PTPN11*, protein tyrosine phosphatase non-receptor type 11; *SF3B2*, splicing factor 3B subunit 2*; SOX9*, SRY-box 9; *SNORD116*, CD box 116; *TCOF1*, treacle ribosome biogenesis factor 1; *TGFBR1*, transforming growth factor-β receptor 1; *TGFBR2*, transforming growth factor-β receptor 2; *TWIST1*, twist family bHLH transcription factor 1; *VPS13B*, vacuolar protein sorting 13 homolog B.

We prepared an overview of possible candidate genes and loci for pediatric SRBD. Figure 2 depicts genes and loci associated both with POSA in children without craniofacial features, and those associated with syndromes manifested by craniofacial features risky for SRBD in children (6–9).

**Figure 3:**
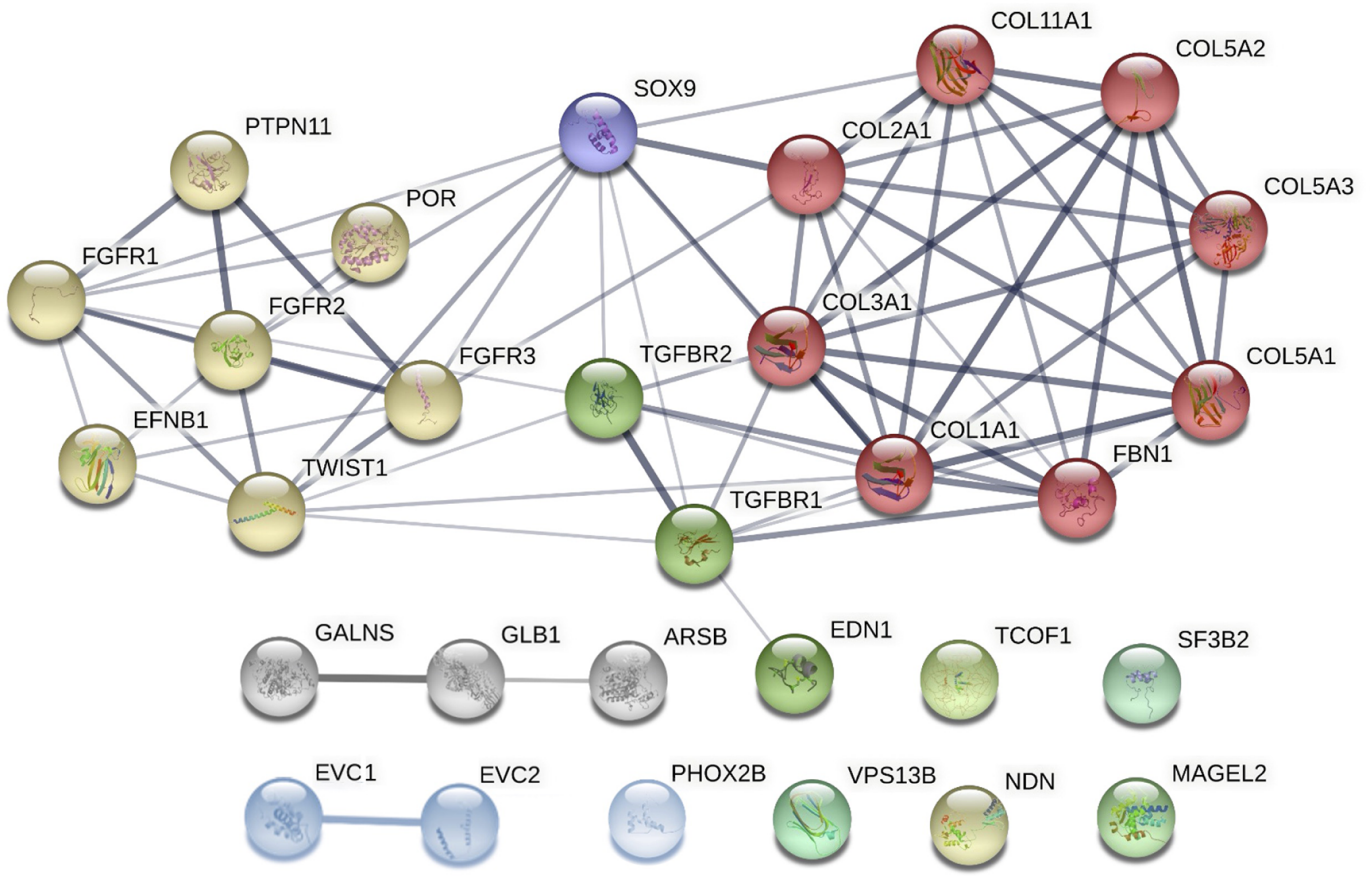
Clusters of candidate genes for pediatric obstructive sleep apnea (POSA) development in children with craniofacial dysmorphisms and their interactions (created in string, https://string-db.org/cgi/network?taskId = bF5B2GAcAUOE&sessionId = bSUqeCIqj6OL). Proteins encoded by these genes are clustered into 3 main groups using the Markov Clustering Algorithm. yellow dots: genes associated with non-/syndromic craniosynostosis. red, purple, and green dots: other genes associated with non-/syndromic retrognathia and/or micrognathia. grey and blue dots: other genes associated with non-/syndromic midface or maxillary hypoplasia. *ARSB,* N-acetylgalactosamine-4 sulfatase; *COL1A1*, collagen type I alpha 1 chain; *COL2A1*, collagen type II alpha 1 chain; *COL3A1*, collagen type III alpha 1 chain; *COL5A1*, collagen type V alpha 1 chain; *COL5A2*, collagen type V alpha 2 chain; *COL5A3*, collagen type V alpha 3 chain; *COL11A1*, collagen type XI alpha 1 chain; *EFNB1*, ephrin-B1*; EDN1*, endothelin 1; *EVC1,* EvC ciliary complex subunit 1*; EVC2*, EvC ciliary complex subunit 2*; FBN1*, fibrillin 1; *FGFR1*, fibroblast growth factor receptor 1; *FGFR2*, fibroblast growth factor receptor 2; *FGFR3*, fibroblast growth factor receptor 3; *GALNS,* galactosamine-6-sulfatase; *GLB1*, b-D-galactosidase; *MAGEL2*, MAGE-like protein 2; *NDN*, necidin; *PHOX2B*, paired like homeobox 2B; POR, cytochrome P450 oxidoreductase; *PTPN11*, protein tyrosine phosphatase non-receptor type 11; *SF3B2*, splicing factor 3B subunit 2*; SOX9*, SRY-box 9; *SNORD116*, CD box 116; *TCOF1*, treacle ribosome biogenesis factor 1; *TGFBR1*, transforming growth factor-β receptor 1; *TGFBR2*, transforming growth factor-β receptor 2; *TWIST1*, twist family bHLH transcription factor 1; *VPS13B*, vacuolar protein sorting 13 homolog B.

Although these syndromes do not share the same genetic background, some of the associated genes affect similar processes, such as the skeletal system development (including the cranial area), organ growth, or embryonic organ morphogenesis. Figure 3 demonstrates both known and predicted interactions and similarities among 30 considered genes; their functions and importance are described below.

### 4.1. Genes associated with non-/syndromic craniosynostosis

The etiology of craniosynostosis may involve genetic, epigenetic, and/or environmental factors (88). Craniosynostosis is associated with a high prevalence of POSA. It is a common feature in patients with Antley-Bixler, Apert, Beare-Stevenson, Crouzon, Pfeiffer, Muenke, Jackson-Weiss, Craniofrontonasal, and Saethre-Chotzen syndromes (89–92). SRDB was present also in 50% of children suffering from non-syndromic craniosynostosis (NSC) (89).

Deviations in the development of the craniofacial area are also associated with a variability in the fibroblast growth factor receptor (*FGFR*) genes, which are important for cell specialization as well as for bone growth and modeling, especially in the process of ossification and bone fusion (48, 93, 94). Severe mutations in *FGFR* genes are associated with premature cranial bone fusion and craniosynostosis. These mutations were found in several craniofacial syndromes with a high prevalence of SRBD in children, such as achondroplasia, Antley-Bixler, Beare-Stevenson, Jackson-Weiss, Apert, Crouzon, Pfeiffer, and Saethre-Chotzen syndromes (21, 51, 92, 95–99). These *FGFR*-related craniosynostosis syndromes are autosomal-dominantly inherited.

Moreover, variants in *FGFR* genes could also lead to NSC (95, 100, 101)*.* Genes most commonly mutated in familial craniosynostosis include, besides *FGFR2* and *FGFR3*, the twist family bHLH transcription factor 1 (*TWIST1*) and ephrin-B1 (*EFNB1*) (102). More than 100 mutations in the *EFNB1* gene have been found to cause the craniofrontonasal syndrome, which was confirmed in a study with knockout mice models (103). This rare x-linked disorder shows paradoxically greater severity in heterozygous females than in hemizygous males. TWIST1 acts through Eph–ephrin interactions to regulate the development of the boundary that forms the coronal suture (104). The *TWIST1* gene associated with the Saethre-Chotzen syndrome is believed to regulate bone formation through other genes, such as *FGFR* and *RUNX2* (63, 64). Genetic testing of *FGFR1, FGFR2, FGFR3*, and *TWIST1* was even suggested as a first-line test for patients with NSC (101).

Interestingly, not only rare mutations of these genes but also SNPs of these genes are associated with craniofacial dysmorphia. For example, Da Fontoura et al. found an association between SNPs rs11200014 and rs2162540 in *FGFR2* and sagittal maxilla-mandibular discrepancy, so-called skeletal malocclusion (both skeletal class II and III). They also found an association between the SNP rs2189000 in *TWIST1* and a larger body and shorter ramus of the mandible (62). Although *FGFR3* gene variants are associated with Muenke and Crouzon syndromes manifested by craniosynostosis, this feature, surprisingly, was not exhibited in the *FGFR3 ^A385E/+^* mice model (105–107). Thus, *FGFR2* seems to be more important for craniosynostosis development than *FGFR3*. On the other hand, a mutation in *FGFR3* causes achondroplasia, which, according to a recent study by Legare et al., has craniosynostosis as a co-occurring feature (108).

A missense mutation in the Protein Tyrosine Phosphatase Non-Receptor Type 11 (***PTPN11***) gene was found in almost 50% of patients diagnosed with Noonan syndrome (109). This gene encodes tyrosine phosphatase Shp-2, an enzyme involved in multiple signal transduction cascades including receptors for growth factors involved in the developmental processes, such as FGFR (110). The Noonan syndrome is manifested by micrognathia, maxillomandibular discrepancy, narrow and/or high-arched palate, and long face syndrome (hyperdivergence) (111, 112). Also, craniosynostosis was described in some patients suffering from this syndrome. Mutations in the *PTPN11*, *KRAS,* or Leucine-Rich Repeat Scaffold Protein (*SHOC2*) gene are causally involved in craniosynostosis (113–115). In patients with the Antley-Bixler syndrome, characterized by craniosynostosis, brachycephaly, midface hypoplasia, and choanal atresia and/or stenosis, variants have been found not only in *FGFR2*, but also in the gene encoding cytochrome p450 oxidoreductase (***POR***) (116–119). This enzyme transfers electrons from NADPH to all microsomal cytochrome P450 enzymes. While individuals with an ABS-like phenotype and normal steroidogenesis are carriers of *FGFR2* mutations, those with genital anomalies and disordered steroidogenesis should be recognized as having a POR deficiency (116).

### 4.2. Genes associated with non-/syndromic retrognathia and/or micrognathia

The SRY-box 9 transcription factor (***SOX9***) gene plays an important regulatory role during craniofacial development (120). In a rat model with upper airway obstruction, SOX9 level was found to be downregulated, which explains the bone architecture abnormalities (121). This gene is also associated with the Pierre Robin sequence (122). Repressed *SOX9* expression leads to changes in the expression of genes essential for normal development of the mandible, causing micrognathia, and, consequently, glossoptosis, airway obstruction, and often, cleft palate (123). The expression of *SOX9* is influenced, among others, by *FGFR3.* Therefore, dysregulation of SOX9 levels, a major regulator of chondrogenesis, is an important underlying mechanism in skeletal diseases caused by mutations in *FGFR3* (124–126). Interestingly, the SNP rs12941170 of *SOX9* was associated with non-syndromic orofacial clefting. However, its role in these non-syndromic clefts remains unclear (124).

Similarly to *SOX9*, variants in endothelin 1 (***EDN1***)*,* the Splicing factor 3B subunit (***SF3B2***)*,* and Treacle ribosome biogenesis factor 1 (***TCOF1***) were associated with syndromes manifesting in children by both micrognathia and glossoptosis. ***EDN1*** encodes a vasoactive peptide belonging to the family of endothelins and is associated with the auriculocondylar syndrome (127), a rare syndrome that usually affects facial features. It is characterized by micrognathia, microstomia, and anomalies in the temporomandibular joint and the condyle (127). Also, studies using mice models with the *EDN1* gene knocked out or deficient have shown several craniofacial dysmorphisms, mandibular dysfunction, and severe retrognathism (62, 127). ***SF3B2*** may be, according to a study by Timberlake et al., an important factor in the development of craniofacial microsomia, which was also confirmed by a recent review covering this congenital facial anomaly (128, 129). ***TCOF1*** presents an important factor for the undisrupted formation and development of the craniofacial area, cartilage, and skeleton (55, 130, 131). Mutations in these genes were found in patients with Treacher-Collins syndrome (130, 131).

Ehlers-Danlos syndrome, manifesting through retrognathia, micrognathia, and maxillary constriction, has been previously proposed as a genetic model for pediatric OSA (60, 61, 132). Variants in genes encoding and/or influencing the expression of collagens (***COL*** gene family) and others (see Supplementary Table S1 in the Supplement) were associated with this rare connective tissue disorder (60, 133). The minor allele of SNP rs2249492 in the Collagen type I alpha 1 chain (***COL1A1***) has been previously associated with the increased risk of a sagittal maxilla-mandibular discrepancy (skeletal class III malocclusion) in non-syndromic children (62). The results of the study by Topârcean et al. showed a tendency towards a class II skeletal malocclusion pattern determined by mandibular retrognathism rather than maxillary prognathism among the individuals possessing the mutant allele of this SNP (134).

Other genes for collagens, ***COL2A1*** and ***COL11A1,*** are associated with Marshall-Stickler syndrome (86). Collagens II and XI are present throughout the Meckel's cartilage, which provides mechanical support for the developing mandible. The characteristic craniofacial features of Marshall-Stickler syndrome are midface hypoplasia, micrognathia, cleft palate, and Pierre Robin anomaly (50). Variants in *COL2A1* and *COL11A1* were also associated with the Robin sequence in nonsyndromic patients (135).

Besides collagens, fibrillin and elastin are also present in the architectural scaffolds that impart specific mechanical properties to tissues and organs. The ***FBN1*** gene is essential for the production of fibrillin, and its mutation could cause Marfan syndrome (57, 136).

Fibrillin is crucial for bone and muscle rigidity; hence, its disruption can increase the laxity of airway connective tissues and predispose them to easier collapsibility (56). At the same time, patients often have their maxillo-mandibular complex in a retrognathic position, with a narrow maxilla and palate, and a “long face” appearance (56–58, 137).

Besides *FBN1*, mutations in the transforming growth factor-β receptor 1 (*TGFBR1)* and transforming growth factor-β receptor 2 (*TGFBR2)* may also be found in Marfan syndrome (138). TGFBR2 protein forms a complex with TGFBR1, and both are involved in a signaling pathway responsible for the proliferation, differentiation, and apoptosis of cells throughout the body (139). They are extremely important for bone growth and extracellular matrix formation; moreover, they play a role in the fusion of craniofacial sutures (140). The development of micrognathia and retrognathism was observed in mice with an impaired *TGFB2* gene, giving evidence to its importance in craniofacial morphology (141).

The gene for vacuolar protein sorting-associated protein 13B (*VPS13B)*, also called the *COH1* gene, encodes a protein forming a part of the Golgi apparatus membrane. Its disruption may be involved through various cellular mechanisms, in several clinical features of Cohen syndrome (142, 143), including micrognathia, constricted hard palate, insufficient lip seal, and truncal obesity. All of these issues increase the risk of the collapse of the upper airway and the development of POSA (65, 66, 142, 143).

Mutations in necidin (*NDN*) and the melanoma antigen family member L2 (*MAGEL2*), both localized on chromosome 15, were found in the Prader-Willi syndrome, a complex genetic disorder characterized by several features, such as midface hypoplasia and micrognathia (144). The phenotype of this syndrome includes hypoplastic midface area, hypotonia, and a changed viscosity in secretions. All these factors facilitate the collapse of upper airways and apnea (52, 53, 144). Some polymorphisms in *NDN* were determined in extremely obese German children and adolescents as well as in neonates examined by polysomnography. However, there was a lack of association with juvenile-onset human obesity or sleep and respiratory parameters (145, 146).

### 4.3. Genes associated with non-/syndromic midface or maxillary hypoplasia

Midface or maxillary hypoplasia are typical features of several syndromes, including mucopolysaccharidosis, Ellis-van Creveld, or congenital central hypoventilation syndrome, the genetic backgrounds of which are described below.

Mucopolysaccharidosis (MPS) is a metabolic disorder characterized by the deficiency or total absence of enzymes responsible for the degradation of glycosaminoglycans. It can be classified into 7 types based on the specific malfunctioning enzyme and clinical manifestations (147). The Morquio syndrome (MPS IV) can be caused by a mutation either in the N-acetylgalactosamine-6-sulfatase (***GALNS***) gene (MPS type IVA), or in the gene for galactosidase beta 1 (***GLB1***; MPS type IVB). Among other clinical manifestations, the Morquio syndrome includes also craniofacial dysmorphisms such as mid-facial hypoplasia, condylar deformities, open bite, macroglossia, or abnormal teeth (148, 149).

The mutated gene for arylsulfatase B (***ARSB***) leads to the reduced function of the enzyme, causing a lysosomal storage disorder – MPS type VI, also known as Maroteaux-Lamy syndrome (117). This syndrome is associated with orofacial manifestations such as macroglossia, malocclusions, or disrupted dental eruption (150).

In syndromic children, the ***TWIST*** gene and the genes of the ***FGFR* family**, described in detail above, were associated with maxillary hypoplasia. In addition, the EvC ciliary complex subunit 1 (***EVC1***) and subunit 2 (***EVC2***) genes were found to be causative for the formation of the Ellis-van Creveld syndrome manifested also by maxillary hypoplasia and mandibular prognathism (151, 152). They encode proteins, the functions of which are not completely understood yet, but appear to be important in the physiological growth and development of bones and teeth (153).

The paired-like homeobox 2B (PHOX2B) transcription factor plays a crucial role in the autonomic nervous system development. Mutations in the ***PHOX2B*** gene are known to cause the congenital central hypoventilation syndrome with a specific craniofacial phenotype – maxillary hypoplasia, box-shaped face, and brachycephaly (68). However, a “silent” mutation in this gene was found in children with class III skeletal malocclusion and a history of sleep apnea (63, 154, 155).

## 5. Discussion

As POSA may cause serious health problems in young, growing patients, it would be highly beneficial to diagnose the increased risk of its development as soon as possible. While much of the POSA etiopathogenesis remains underexplored, craniofacial dysmorphisms leading to the narrowing of the airways undoubtedly play an important role (15, 17). Their severe forms can be found in craniofacial syndromes, which are also associated with a much higher prevalence of SRBD and POSA compared to the general pediatric population (21, 45, 53, 54, 56, 58, 63, 69–87). However, similar craniofacial features may be present also in healthy, non-syndromic patients. These skeletal variations could be mild when compared to syndromic phenotypes, but they could still lead to the collapse of the upper airways and POSA development. This is supported by Kim et al. who reported that the majority of non-syndromic, non-obese children diagnosed with POSA have craniofacial anomalies that are possible risk factors for POSA (18).

Several studies have already explored genes associated with OSA etiopathogenesis in adults, including the genes associated with the craniofacial area and characteristic features (4, 156). The heritability of craniofacial traits varies but is generally estimated to be high and very similar in healthy subjects and in patients suffering from OSA (11, 13, 46, 157). This is supported by several studies reporting an increased incidence of the above-mentioned OSA risk features among relatives (157–160). The first phenome-wide association study of genomic variation in adult OSA was recently published by Veatch et al. (5). None of the three SNPs in the leptin receptor (*LEPR*), the matrix metallopeptidase 9 (*MMP9*), and the Gamma-aminobutyric acid type B receptor subunit 1 (*GABBR1*), the association of which with OSA diagnosis was validated in their study, was associated with other non-OSA clinical traits once they controlled for multiple testing (5).

Cade at al. performed a GWAS investigating genetic associations of OSA in Hispanic/Latino Americans from three cohorts. They identified two loci (rs11691765 in the G protein-coupled receptor 83 gene, *GPR83*; and rs35424364 in the pseudogene *CCDC162P*) associated with the AHI and the respiratory event duration, respectively (3). Another GWAS study, focusing on European Caucasians, reported five genes to be associated with facial characteristics, namely the paired-box gene 3 (*PAX3*), the PR-set domain 16 (*PRDM16*), the transcription factor *TP63*, small integral membrane protein 23 (*C5orf50*)*,* and the Collagen type XVII alpha 1 chain (*COL17A1A*), the variants of which contribute to the facial morphology in young adults (4). Some variants of these genes and their possible association with craniofacial abnormalities were also explored in another GWAS study focusing on young adults of European-ancestry from the Avon Longitudinal Study of Parents and Children (161) as well as in mice models (162, 163).

Unfortunately, most publications focus on the genetic background of OSA in adult patients, not the pediatric population. To this date, only one GWAS has been performed in relation to POSA, including European American and African American children without craniofacial disorders (6). The study identified several genomic loci (see the Introduction). However, only one genetic marker, located at 18p11.32, was shared by groups of both ancestries. Their study, therefore, emphasizes the importance of study populations with diverse ethnic backgrounds to identify unique and shared genetic markers that contribute to the heterogeneity of POSA (6).

It follows that specific genes involved in the development of the orofacial area and associated with craniofacial OSA features should be also considered as candidate genes for POSA. Here, we provide an overview of genes that are known to be involved in the development of craniofacial syndromes in children with high SRBD prevalence, including POSA, see Supplementary Table S1 in the Supplement. All these genes are, to some extent, involved in the formation of tissues of the orofacial area. The candidate genes for POSA can be classified into three major groups based on their involvement in the development of specific craniofacial features. These groups would consist of genes associated with non-/syndromic (i) craniosynostosis, (ii) retrognathia and/or micrognathia, and (iii) midface or maxillary hypoplasia. While certain mutations cause various rare syndromes, other variants in these same genes were suggested to be associated with non-syndromic skeletal variations in the orofacial area (62, 63, 101, 154, 164). So far, variants in ***FGFR1****, **FGFR2**, **FGFR3***, ***TWIST, SOX9****, **COL1A1***, and ***PHOX2B*** are known to play a role in syndrome development as well as in the development of skeletal malocclusions (sagittal maxillo-mandibular complex discrepancies in non-syndromic patients). These genes, therefore, can be considered promising candidate genes for testing of genetic susceptibility to POSA development in various populations.

Although the inheritance pattern of POSA as well as OSA is unclear, most cases with these diseases do not adhere to classical models of inheritance, suggesting that multiple genes could be involved in their development. We believe that besides the GWAS approach, strategies based on candidate genes are also necessary for further research of both these multifactorial diseases. Considering the results of the mentioned genetic association studies (3–9) it appears that there is not much overlap between candidate variants/genes for the POSA and OSA development. In addition, these studies also revealed a high interpopulation variability that should be taken into account in the further research of these disorders. The low match in candidate genes for OSA between children and adults is to be expected since those diseases differ in their etiopathogenesis, clinical presentation as well as polysomnographic characteristics; there are also major differences in therapy approaches and possible consequences if left untreated (165).

Recently, Yoon et al. proposed a clinical guideline for application of multidisciplinary care in children with SRBD, emphasizing the importance of dentofacial interventions that target variable growth patterns (166). In the last years, craniofacial modification by orthodontic techniques is increasingly incorporated into the multidisciplinary management of SRBD in children and adolescents. In view of the multifactorial etiology of POSA, a better understanding of the risk factors contributing to its development may be useful not only for predicting the risk of POSA development but, even more importantly, for selecting the best therapeutic approach. Research of genetic predispositons to OSA in children as well as in adults may improve our understanding of the underlying biological mechanisms of susceptibility to these diseases.

## 6. Conclusion

Genetic background plays an important role in both POSA and craniofacial dysmorphisms. Therefore, genes associated with specific craniofacial features more common in patients suffering from POSA may be also considered candidate genes for this disease. We have reviewed a large body of literature and focused on the genes known to be involved in the development of cranio-facial syndromes with a high POSA prevalence. Based on the review, we chose 30 candidate genes for pediatric SRBD. Variants in seven of them (***FGFR1****, **FGFR2**, **FGFR3***, ***TWIST, SOX9****, **COL1A1***, and ***PHOX2B***) are known to play a role not only in syndrome development but also in skeletal malocclusions that are typical of pediatric orthodontic patients. Considering this, these seven genes appear to have the highest potential for targeted analysis of POSA risk in non-syndromic children.
