# Supplementary material for: Candidate genes for obstructive sleep apnea in non-syndromic children with craniofacial dysmorphisms – a narrative review
Source: Front Pediatr. 2023 Jun 27;11:1117493. doi: 10.3389/fped.2023.1117493 (PMC10334820; doi:10.3389/fped.2023.1117493)
Supplement: Supplementary file 1 [file Table1.pdf]

Supplementary Table 1. Genetic background of syndromes/disorders associated with both craniofacial dysmorphisms and high prevalence of pediatric SRBD (sleep-related breathing disorder), including pediatric obstructive sleep apnea

**Syndrome/Disorder**	**Prevalence of SRBD**	**Craniofacial dysmorphisms potentially limiting the airway patency**	**Associated genes or aneuploidy**
Achondroplasia	10.0-75.0% (1, 2)	midface hypoplasia, maxillary hypoplasia, retruded chin, increased mandibular plane angle (3-5)	*FGFR3* (6)
Antley-Bixler	unknown (7)	craniosynostosis, brachycephaly, midface hypoplasia, choanal atresia, stenosis (7, 8)	*FGFR2, FGFR3, POR* (9, 10)
Apert	80.6% (11)	craniosynostosis, midface hypoplasia, cleft palate (12, 13)	*FGFR2* (12, 14, 15)
Auriculocondylar	unknown (16)	micrognathia, retrognathia, cleft palate, glossoptosis, choanal stenosis (17, 18)	*EDN1* (19)
Beare-Stevenson	unknown (20)	craniosynostosis, midface hypoplasia, choanal atresia/stenosis, cleft palate (13, 20)	*FGFR2* (20)
Cohen	unknown (21)	micrognathia, narrow and/or high-arched palate (22, 23)	*VPS13B/COH1* (24)
Congenital central hypoventilation syndrome	unknown (25)	maxillary hypoplasia, brachycephaly (26)	*PHOX2B* (26)
Craniofacial microsomia (Goldenhar syndrome, oculo-auriculo-vertebral spectrum)	unknown (27-29)	hemifacial microsomia, micrognathia, glossoptosis, cleft (28-30)	*SF3B2* (31, 32)

Craniofrontonasal	unknown (33)	craniosynostosis, retrognathia (33, 34)	*EFNB1* (35)
Crouzon	64.7-66.7% (11)	craniosynostosis, maxillary hypoplasia, narrow and/or high-arched palate, micrognathia (11, 12, 36, 37)	*FGFR2, FGFR3* (12, 14, 15, 37, 38)
Down	24.0-59.0% (39-41)	midface hypoplasia, hypoplastic maxilla, micrognathia, narrow and/or high-arched palate, macroglossia (39, 42, 43)	Trisomy of chromosome 21 (39, 44)
Ehlers-Danlos	26.0-42.0% (45-47)	maxillary constriction, retrognathia, micrognathia (48-50)	*e.g.: COL1A1, COL3A1 COL5A1, COL5A2, COL5A3* (48, 49, 51-53)
Ellis-van Creveld	unknown (4)	maxillary hypoplasia, prognathism (54)	*EVC1, EVC2* (55, 56)
Jackson-Weiss	unknown (57)	craniosynostosis (13)	*FGFR2* (58)
Marfan	57.1-72.3% (47, 59)	retrognathia, narrow and/or high-arched palate (47, 59-61)	*FBN1, TGFBR1, TGFRB2* (60, 62)
Marshall-Stickler	unknown (63-65)	midface hypoplasia micrognathia, cleft palate (63, 65, 66)	*COL2A1, COL11A1* (67-69)

Mucopolysaccharidosis IV (Morquio syndrome); Mucopolysaccharidosis VI (Maroteaux – Lamy syndrome)	>80.0% (70, 71)	midface hypoplasia, macroglossia, hyperdivergent (long face) (70-72)	*GALNS, GLB1, ARSB* (70, 71, 73)
Muenke	unknown (74)	craniosynostosis, midface hypoplasia, narrow and/or high-arched palate (13, 75)	*FGFR3* (3, 75)
Noonan	unknown (4)	micrognathia, maxillomandibular discrepancy, narrow and/or high-arched palate, long face (hyperdivergence) (76, 77)	*PTPN11* (78)
Pfeiffer	72.7% (11)	craniosynostosis, maxillary hypoplasia, choanal atresia (14, 79)	*FGFR2, FGFR1* (14, 15)
Pierre Robin sequence	47.0% (80)	micrognathia, glossoptosis, narrow and/or high-arched palate, cleft (4, 81-83)	*SOX9* (84, 85)
Prader-Willi	79.9% (86, 87)	midface hypoplasia, micrognathia (86, 88)	*NDN, MAGEL2* (89)
Saethre-Chotzen	unknown (90)	craniosynostosis, midface hypoplasia, maxillary hypoplasia, long face (hyperdivergence) (21, 91-93)	*TWIST1* (21)
Treacher-Collins	54.0-87.5% (94, 95)	midface hypoplasia, micrognathia, glossoptosis, may include cleft palate and choanal atresia (94, 96, 97)	*TCOF1* (98)

ARSB, N-acetylgalactosamine-4 sulfatase; *COL1A1*, Collagen type I alpha 1 chain; *COL2A1,* Collagen type II alpha 1 chain*; COL3A1,* Collagen type III alpha 1 chain; *COL5A1*, Collagen type V alpha 1 chain; *COL5A2*, Collagen type V alpha 2 chain; *COL5A3*, Collagen type V alpha 3 chain; *COL11A1,* Collagen type XI alpha 1 chain; *EFNB1*, ephrin-B1*; EDN1*, Endothelin 1; *EVC1,* EvC Ciliary Complex Subunit 1*; EVC2,* EvC Ciliary Complex Subunit 2*; FBN1*, Fibrillin 1; *FGFR1*, Fibroblast growth factor receptor 1; *FGFR2*, Fibroblast growth factor receptor 2; *FGFR3*, Fibroblast growth factor receptor 3; *GALNS,* Galactosamine-6-sulfatase; *GLB1*, b-D-galactosidase; *MAGEL2*, MAGE-like protein 2; *NDN*, Necidin; *PHOX2B*, Paired like homeobox 2B; POR, Cytochrome P450 Oxidoreductase; *PTPN11*, Protein Tyrosine Phosphatase Non-Receptor Type 11; *SF3B2*, Splicing factor 3B subunit 2*; SOX9*, SRY-box 9; *SNORD116*, CD box 116; *TCOF1*, Treacle ribosome biogenesis factor 1; *TGFBR1*, Transforming growth factor-β receptor 1; *TGFBR2*, Transforming growth factor-β receptor 2; *TWIST1*, Twist family bHLH transcription factor 1; *VPS13B*, Vacuolar protein sorting 13 homolog B

References

1. Afsharpaiman S, Saburi A, Waters KA. Respiratory Difficulties and Breathing Disorders in Achondroplasia. Paediatr Respir Rev (2013) 14:250-5. doi: 10.1016/j.prrv.2013.02.009.

2. Onodera K, Niikuni N, Chigono T, Nakajima I, Sakata H, Motizuki H. Sleep Disordered Breathing in Children with Achondroplasia: Part 2. Relationship with Craniofacial and Airway Morphology. Int J Pediatr Otorhinolaryngol (2006) 70:453-61. doi: 10.1016/j.ijporl.2005.07.016.

3. Vajo Z, Francomano CA, Wilkin DJ. The Molecular and Genetic Basis of Fibroblast Growth Factor Receptor 3 Disorders: The Achondroplasia Family of Skeletal Dysplasias, Muenke Craniosynostosis, and Crouzon Syndrome with Acanthosis Nigricans. Endocr Rev (2000) 21:23-39. doi: 10.1210/edrv.21.1.0387.

4. Zaffanello M, Antoniazzi F, Tenero L, Nosetti L, Piazza M, Piacentini G. Sleep-Disordered Breathing in Paediatric Setting: Existing and Upcoming of the Genetic Disorders. Ann Transl Med (2018) 6:343. doi: 10.21037/atm.2018.07.13.

5. Zucconi M, Weber G, Castronovo V, Ferini-Strambi L, Russo F, Chiumello G, et al. Sleep and Upper Airway Obstruction in Children with Achondroplasia. J Pediatr (1996) 129:743-9. doi: 10.1016/s0022-3476(96)70159-2.

6. Horton WA, Lunstrum GP. Fibroblast Growth Factor Receptor 3 Mutations in Achondroplasia and Related Forms of Dwarfism. Rev Endocr Metab Disord (2002) 3:381-5. doi: 10.1023/a:1020914026829.

7. Kwon YS, Jo JK, Lim YH, Yon JH, Kim KM. Anesthetic Management of a Neonate with Antley-Bixler Syndrome: A Case Report. Anesthesia and Pain Medicine (2011) 6:89-92.

8. Antley R. Trapezoidocephaly, Midfacial Hypoplasia and Cartilage Abnormalities with Multiple Synostoses and Skeletal Fractures. Birth Defects Orig Artic Ser (1975) 11:397-401.

9. Huang N, Pandey AV, Agrawal V, Reardon W, Lapunzina PD, Mowat D, et al. Diversity and Function of Mutations in P450 Oxidoreductase in Patients with Antley-Bixler

Syndrome and Disordered Steroidogenesis. Am J Hum Genet (2005) 76:729-49. doi: 10.1086/429417.

10. Li H, Zhao A, Xie M, Chen L, Wu H, Shen Y, et al. Antley-Bixler Syndrome Arising from Compound Heterozygotes in the P450 Oxidoreductase Gene: A Case Report. Transl Pediatr (2021) 10:3309-18. doi: 10.21037/tp-21-499.

11. Inverso G, Brustowicz K, Katz E, Padwa B. The Prevalence of Obstructive Sleep Apnea in Symptomatic Patients with Syndromic Craniosynostosis. Int J Orral Maxillofac Surg (2016) 45:167-9. doi: 10.1016/j.ijom.2015.10.003

12. Carinci F, Pezzetti F, Locci P, Becchetti E, Carls F, Avantaggiato A, et al. Apert and Crouzon Syndromes: Clinical Findings, Genes and Extracellular Matrix. J Craniofac Surg (2005) 16:361-8.. doi: 10.1097/01.scs.0000157078.53871.11.

13. Wenger T, Miller D, Evans K. Fgfr Craniosynostosis Syndromes Overview. In: Adam MP, Everman DB, Mirzaa GM, Pagon RA, Wallace SE, Bean LJH, et al., editors. Genereviews*(®)*. [Internet]. Seattle (WA): University of Washington, Seattle; 1993

14. Lajeunie E, Heuertz S, El Ghouzzi V, Martinovic J, Renier D, Le Merrer M, et al. Mutation Screening in Patients with Syndromic Craniosynostoses Indicates That a Limited Number of Recurrent Fgfr2 Mutations Accounts for Severe Forms of Pfeiffer Syndrome. Eur J Hum Genet. (2006) 14:289-98. doi: 10.1038/sj.ejhg.5201558.

15. Azoury SC, Reddy S, Shukla V, Deng CX. Fibroblast Growth Factor Receptor 2 (Fgfr2) Mutation Related Syndromic Craniosynostosis. Int J Biol Sci (2017) 13:1479-88. doi: 10.7150/ijbs.22373.

16. Storm AL, Johnson JM, Lammer E, Green GE, Cunniff C. Auriculo-Condylar Syndrome Is Associated with Highly Variable Ear and Mandibular Defects in Multiple Kindreds. Am J Med Genet A (2005) 138:141-5. doi: 10.1002/ajmg.a.30883.

17. Kurihara Y, Kurihara H, Suzuki H, Kodama T, Maemura K, Nagai R, et al. Elevated Blood Pressure and Craniofaclal Abnormalities in Mice Deficient in Endothelin-1. Nature (1994) 368:703-10. doi: 10.1038/368703a0.

18. Peart LS, Gonzalez J, Bivona S, Latchman K, Torres L, Network UD, et al. Bilateral Choanal Stenosis in Auriculocondylar Syndrome Caused by a Plcb4 Variant. Am J Med Genet A (2022) 188:1307-10. doi: 10.1002/ajmg.a.62634.

19. Gordon CT, Petit F, Kroisel PM, Jakobsen L, Zechi-Ceide RM, Oufadem M, et al. Mutations in Endothelin 1 Cause Recessive Auriculocondylar Syndrome and Dominant Isolated Question-Mark Ears. Am J Hum Genet (2013) 93:1118-25. doi: 10.1016/j.ajhg.2013.10.023.

20. Slavotinek A, Crawford H, Golabi M, Tao C, Perry H, Oberoi S, et al. Novel Fgfr2 Deletion in Patient with Beare-Stevenson-Like Syndrome. Am J Med Genet A (2009) 149:1814. doi: 10.1002/ajmg.a.32947.

21. Hartsfield Jr JK, Morford LA, Jacob GJ, Kluemper GT. Genetic Factors Affecting Facial Morphology Associated with Sleep Apnea.

22. Cohen Jr MM. A New Syndrome with Hypotonia, Obesity, Mental Deficiency, and Facial, Oral, Ocular, and Limb Anomalies. J Pediatr(1973) 83:280-4. doi: 10.1016/s0022-3476(73)80493-7.

23. Chandler K, Kidd A, Al-Gazali L, Kolehmainen J, Lehesjoki A-E, Black GC, et al. Diagnostic Criteria, Clinical Characteristics, and Natural History of Cohen Syndrome. J Med Genet (2003) 40:233-41. doi: 10.1136/jmg.40.4.233.

24. Duplomb L, Duvet S, Picot D, Jego G, El Chehadeh-Djebbar S, Marle N, et al. Cohen Syndrome Is Associated with Major Glycosylation Defects. Hum Mol Genet (2014) 23:2391-9. doi: 10.1093/hmg/ddt630.

25. Wang P, Zhou W, Yuan W, Huang L, Zhao N, Chen X. Prader–Willi Syndrome in Neonates: Twenty Cases and Review of the Literature in Southern China. BMC Pediatr (2016) 16:1-6 doi: 10.1186/s12887-016-0662-2.

26. Todd ES, Weinberg SM, Berry-Kravis EM, Silvestri JM, Kenny AS, Rand CM, et al. Facial Phenotype in Children and Young Adults with Phox2b-Determined Congenital Central Hypoventilation Syndrome: Quantitative Pattern of Dysmorphology. Pediatr Res (2006) 59:39-45. Epub 2005/12/06. doi: 10.1203/01.pdr.0000191814.73340.1d.

27. Baugh A, Wooten W, Chapman B, Drake A, Vaughn B. Sleep Characteristics in Goldenhar Syndrome. Int J Pediatf Otorhinolaryngol (2015) 79:356-8. doi: 10.1016/j.ijporl.2014.12.024.

28. Kourelis K, Gouma P, Naxakis S, Kalogeropoulou C, Goumas P. Oculoauriculovertebral Complex with an Atypical Cause of Obstructive Sleep Apnea. Int J Pediatr Otorhinolaryngol (2009) 73:481-5. doi: 10.1016/j.ijporl.2008.11.004.

29. Abraham C, Virbalas J, DelRosso LM. Severe Obstructive Sleep Apnea in a Child with Goldenhar Syndrome and Nasal Obstruction. J Clin Sleep Med (2017) 13:825-7. doi: 10.5664/jcsm.6626.

30. Beleza-Meireles A, Hart R, Clayton-Smith J, Oliveira R, Reis CF, Venâncio M, et al. Oculo-Auriculo-Vertebral Spectrum: Clinical and Molecular Analysis of 51 Patients. Eur J Med Genet (2015) 58:455-65. doi: 10.1016/j.ejmg.2015.07.003.

31. Timberlake AT, Griffin C, Heike CL, Hing AV, Cunningham ML, Chitayat D, et al. Haploinsufficiency of Sf3b2 Causes Craniofacial Microsomia. Nat Commun (2021) 12:1-11. doi: 10.1038/s41467-021-24852-9.

32. Tingaud-Sequeira A, Trimouille A, Sagardoy T, Lacombe D, Rooryck C. Oculo-Auriculo-Vertebral Spectrum: New Genes and Literature Review on a Complex Disease. J Med Genet (2022) 59:417-27. doi: 10.1136/jmedgenet-2021-108219.

33. Verstraete L, Shaheen E, Grymonprez E, Miclotte I, Politis C. Chin Wing Osteotomy in a Patient with Craniofrontonasal Dysplasia. Oral Maxillofac Surg Cases (2020) 6:100157.

34. Zafeiriou DI, Pavlidou EL, Vargìami E. Diverse Clinical and Genetic Aspects of Craniofrontonasal Syndrome. Pediatr Neurol (2011) 44:83-7. doi: 10.1016/j.pediatrneurol.2010.10.012.

35. Twigg SR, Kan R, Babbs C, Bochukova EG, Robertson SP, Wall SA, et al. Mutations of Ephrin-B1 (Efnb1), a Marker of Tissue Boundary Formation, Cause Craniofrontonasal Syndrome. Proc Natl Acad Sci U S A (2004) 101:8652-7. doi: 10.1073/pnas.0402819101.

36. Driessen C, Joosten KF, Bannink N, Bredero-Boelhouwer HH, Hoeve HL, Wolvius EB, et al. How Does Obstructive Sleep Apnoea Evolve in Syndromic Craniosynostosis? A

Prospective Cohort Study. Arch Dis Child (2013) 98:538-43. doi: 10.1136/archdischild-2012-302745.

37. Khominsky A, Yong R, Ranjitkar S, Townsend G, Anderson PJ. Extensive Phenotyping of the Orofacial and Dental Complex in Crouzon Syndrome. Arch Oral Biol (2018) 86:123-30. doi: 10.1016/j.archoralbio.2017.10.022.

38. Cornille M, Moriceau S, Khonsari RH, Heuzé Y, Loisay L, Boitez V, et al. Fgfr3 Overactivation in the Brain Is Responsible for Memory Impairments in Crouzon Syndrome Mouse Model. J Exp Med (2022) 219. doi: 10.1084/jem.20201879.

39. Churchill SS, Kieckhefer GM, Landis CA, Ward TM. Sleep Measurement and Monitoring in Children with Down Syndrome: A Review of the Literature, 1960–2010. Sleep Med Rev (2012) 16:477-88. doi: 10.1016/j.smrv.2011.10.003.

40. Ng DK, Chan C-H. Obesity Is an Important Risk Factor for Sleep Disordered Breathing in Children with Down Syndrome. Sleep (2004) 27:1023-4; author reply 5.

41. Dahlqvist Å, Rask E, Rosenqvist C-J, Sahlin C, Franklin KA. Sleep Apnea and Down's Syndrome. Acta Otolaryngol (2003) 123:1094-7. doi: 10.1080/00016480310015362.

42. Cielo CM, Konstantinopoulou S, Hoque R. Osas in Specific Pediatric Populations. Curr Probl Pediatr Adolesc Health Care (2016) 1:11-8. doi: 10.1016/j.cppeds.2015.10.008.

43. Macho V, Coelho A, Areias C, Macedo P, Andrade D. Craniofacial Features and Specific Oral Characteristics of Down Syndrome Children. Oral Health Dent Manag (2014) 13:408-11.

44. Shott SR, Amin R, Chini B, Heubi C, Hotze S, Akers R. Obstructive Sleep Apnea: Should All Children with Down Syndrome Be Tested? Arch Otolaryngol Head Neck Surg (2006) 132:432-6. doi: 10.1001/archotol.132.4.432.

45. Stöberl AS, Gaisl T, Giunta C, Sievi NA, Singer F, Möller A, et al. Obstructive Sleep Apnoea in Children and Adolescents with Ehlers-Danlos Syndrome. Respiration (2019) 97:284-91. doi: 10.1159/000494328.

46. Domany KA, Hantragool S, Smith DF, Xu Y, Hossain M, Simakajornboon N. Sleep Disorders and Their Management in Children with Ehlers-Danlos Syndrome Referred to Sleep Clinics. J Clin Sleep Med (2018) 14:623-9. doi: 10.5664/jcsm.7058

47. Sedky K, Gaisl T, Bennett DS. Prevalence of Obstructive Sleep Apnea in Joint Hypermobility Syndrome: A Systematic Review and Meta-Analysis. J Clin Sleep Med (2019) 15:293-9. doi: 10.5664/jcsm.7636.

48. Guilleminault C, Primeau M, Chiu HY, Yuen KM, Leger D, Metlaine A. Sleep-Disordered Breathing in Ehlers-Danlos Syndrome: A Genetic Model of Osa. Chest (2013) 144:1503-11. doi: 10.1378/chest.13-0174.

49. Malfait F, Wenstrup RJ, De Paepe A. Clinical and Genetic Aspects of Ehlers-Danlos Syndrome, Classic Type. Genet Med (2010) 12:597-605. doi: 10.1097/GIM.0b013e3181eed412.

50. Van Camp N, Aerden T, Politis C. Problems in the Orofacial Region Associated with Ehlers-Danlos and Marfan Syndromes: A Case Series. Br J Oral Maxillofac Surg (2020) 58:208-13. doi: 10.1016/j.bjoms.2019.11.018.

51. De Paepe A, Malfait F. The Ehlers-Danlos Syndrome, a Disorder with Many Faces. Clin Genet (2012) 82:1-11. doi: 10.1111/j.1399-0004.2012.01858.x.

52. Omar R, Malfait F, Van Agtmael T. Four Decades in the Making: Collagen Iii and Mechanisms of Vascular Ehlers Danlos Syndrome. Matrix Biology Plus (2021) 12:100090. doi: 10.1016/j.mbplus.2021.100090.

53. Van Damme T, Colman M, Syx D, Malfait F. The Ehlers-Danlos Syndromes against the Backdrop of Inborn Errors of Metabolism. Genes (Basel) (2022) 13. doi: 10.3390/genes13020265.

54. Kalaskar R, Kalaskar AR. Oral Manifestations of Ellis-Van Creveld Syndrome. Contemp Clin Dent (2012) 3:S55. doi: 10.4103/0976-237X.95106.

55. Galdzicka M, Patnala S, Hirshman M, Cai J-F, Nitowsky H, Egeland J, et al. A New Gene, Evc2, Is Mutated in Ellis–Van Creveld Syndrome. Mol Genet Metab (2002) 77:291-5. doi: 10.1016/s1096-7192(02)00178-6.

56. Ruiz-Perez VL, Ide SE, Strom TM, Lorenz B, Wilson D, Woods K, et al. Mutations in a New Gene in Ellis-Van Creveld Syndrome and Weyers Acrodental Dysostosis. Nat Genet (2000) 24:283-6. doi: 10.1038/73508.

57. Gasparini G, Di Rocco C, Saponaro G, Marianetti TM, Foresta E, Rinaldo FMD, et al. Evaluation of Obstructive Sleep Apnea in Pediatric Patients with Facio-Craniostenosis: A Brief Communication. Childs Nerv Syst (2012) 28:1135-40. doi: 10.1007/s00381-012-1821-x.

58. Tartaglia M, Di Rocco C, Lajeunie E, Valeri S, Velardi F, Battaglia PA. Jackson-Weiss Syndrome: Identification of Two Novel Fgfr2 Missense Mutations Shared with Crouzon and Pfeiffer Craniosynostotic Disorders. Hum Genet (1997) 101:47-50. doi: 10.1007/s004390050584.

59. Cistulli PA, Sullivan CE. Sleep Apnea in Marfan's Syndrome: Increased Upper Airway Collapsibility During Sleep. Chest (1995) 108:631-5. doi: 10.1378/chest.108.3.631.

60. De Coster P, Pauw GD, Martens L, De Paepe A. Craniofacial Structure in Marfan Syndrome: A Cephalometric Study. Am J Med Genet A (2004) 131:240-8. doi: 10.1002/ajmg.a.30393.

61. Docimo R, Maturo P, D'Auria F, Grego S, Costacurta M, Perugia C, et al. Association between Oro-Facial Defects and Systemic Alterations in Children Affected by Marfan Syndrome. J. Clin Diagn Res (2013) 7:700-3. doi: 10.7860/JCDR/2013/5656.2885.

62. Akutsu K, Morisaki H, Takeshita S, Sakamoto S, Tamori Y, Yoshimuta T, et al. Phenotypic Heterogeneity of Marfan-Like Connective Tissue Disorders Associated with Mutations in the Transforming Growth Factor-B Receptor Genes. Circ J (2007) 71:1305-9. doi: 10.1253/circj.71.1305.

63. LaPenna R, Folger Jr GM. Extreme Upper Airway Obstruction with the Marshall Syndrome. Clin Pediatr (1982) 21:507-10. doi: 10.1177/000992288202100815.

64. Miloro M. Mandibular Distraction Osteogenesis for Pediatric Airway Management. J Oral Maxillofac Surg (2010) 68:1512-23. doi: 10.1016/j.joms.2009.09.099.

65. Cielo CM, Marcus CL. Obstructive Sleep Apnoea in Children with Craniofacial Syndromes. Paediatr Respir Rev. (2015) 16:189-96.

66. Janssen EJ, Stegmann AP, Stumpel CT. Distinguishing Marshall from Stickler Syndrome: A Clinical and Genetic Challenge. Clin Dysmorphol (2021) 30:58-61. doi: 10.1097/MCD.0000000000000346

67. Snead MP, Yates JR. Clinical and Molecular Genetics of Stickler Syndrome. J Med Genet (1999) 36:353-9.

68. Mladenova M, Todorov T, Grozdanova L, Mitev V, Todorova A. Novel Mutation in the Gene Causing Marshall-Stickler Syndrome in Three Generations of a Bulgarian Family. Balkan J Med Genet (2021) 24:95-8. doi: 10.2478/bjmg-2021-0001.

69. Guo L, Elcioglu NH, Wang Z, Demirkol YK, Isguven P, Matsumoto N, et al. Novel and Recurrent Col11a1 and Col2a1 Mutations in the Marshall–Stickler Syndrome Spectrum. Hum Genome Var (2017) 4:1-4. doi: 10.1038/hgv.2017.40.

70. Berger KI, Fagondes SC, Giugliani R, Hardy KA, Lee KS, McArdle C, et al. Respiratory and Sleep Disorders in Mucopolysaccharidosis. J Inherit Metab Dis (2013) 36:201-10. doi: 10.1007/s10545-012-9555-1.

71. Pal AR, Brown N, Jones SA, Bigger BW, Bruce IA. Obstructive Sleep Apnea in Mps: A Systematic Review of Pretreatment and Posttreatment Prevalence and Severity. J Inborn Errors Metab Screen (2019) 3. doi:10.1177/2326409815616392.

72. Ribeiro EM, Fonteles CS, Freitas AB, da Silva Alves KS, Monteiro AJ, Bruno da Silva CA. A Clinical Multicenter Study of Orofacial Features in 26 Brazilian Patients with Different Types of Mucopolysaccharidosis. Cleft Palate Craniofac J (2015) 52:352-8. doi: 10.1597/13-204.

73. Garrido E, Cormand B, Hopwood JJ, Chabás A, Grinberg D, Vilageliu L. Maroteaux-Lamy Syndrome: Functional Characterization of Pathogenic Mutations and Polymorphisms in the Arylsulfatase B Gene. Mol Genet Metab (2008) 94:305-12. doi: 10.1016/j.ymgme.2008.02.012

74. Bannink N, Maliepaard M, Raat H, Joosten KF, Mathijssen IM. Obstructive Sleep Apnea-Specific Quality of Life and Behavioral Problems in Children with Syndromic Craniosynostosis. J Dev Behav Pediatr (2011) 32:233-8. doi: 10.1097/DBP.0b013e318206d5e3.

75. Agochukwu NB, Solomon BD, Doherty ES, Muenke M. Palatal and Oral Manifestations of Muenke Syndrome (Fgfr3-Related Craniosynostosis). J Craniofac Surg (2012) 23:664-8. doi: 10.1097/SCS.0b013e31824db8bb.

76. Allanson JE. Noonan Syndrome. J Med Genet (1987) 24:9. doi: 10.1136/jmg.24.1.9.

77. Cardiel Ríos SA. Correction of a Severe Class Ii Malocclusion in a Patient with Noonan Syndrome. Am J Orthod Dentofacial Orthop (2016) 150:511-20. doi: 10.1016/j.ajodo.2015.09.032.

78. van der Burgt I. Noonan Syndrome. Orphanet J Rare Dis (2007) 2:4. doi: 10.1186/1750-1172-2-4.

79. Oyamada MK, Ferreira HSA, Hoff M. Pfeiffer Syndrome Type 2: Case Report. Sao Paulo Med J (2003) 121:176-9. doi: 10.1590/s1516-31802003000400008.

80. Khayat A, Bin-Hassan S, Al-Saleh S. Polysomnographic Findings in Infants with Pierre Robin Sequence. Ann Thor Med (2017) 12:25-9. doi: 10.4103/1817-1737.197770.

81. Robin P. Glossoptosis Due to Atresia and Hypotrophy of the Mandible. Am J Dis Child (1934) 48:541-7.

82. Gangopadhyay N, Mendonca DA, Woo AS, editors. Pierre Robin Sequence. Semin Plast Surg (2012) 26:76-82. doi: 10.1055/s-0032-1320065.

83. Rampova S, Urbanova W, Langova K, Kotova M. Growth of the mandible in patients with Pierre Robin sequence. Ortodoncie, 2022, 31(4): 277-287.

84. Benko S, Fantes JA, Amiel J, Kleinjan D-J, Thomas S, Ramsay J, et al. Highly Conserved Non-Coding Elements on Either Side of Sox9 Associated with Pierre Robin Sequence. Nature genetics (2009) 41:359-64. doi: 10.1016/j.prrv.2014.11.003.

85. Gordon CT, Attanasio C, Bhatia S, Benko S, Ansari M, Tan TY, et al. Identification of Novel Craniofacial Regulatory Domains Located Far Upstream of Sox 9 and Disrupted in Pierre Robin Sequence. Hum Mutat (2014) 35:1011-20. doi: 10.1002/humu.22606.

86. Sedky K, Bennett DS, Pumariega A. Prader Willi Syndrome and Obstructive Sleep Apnea: Co-Occurrence in the Pediatric Population. J Clin Sleep Med (2014) 10:403-9. doi: 10.5664/jcsm.3616.

87. Lin HY, Lin SP, Lin CC, Tsai LP, Chen MR, Chuang CK, et al. Polysomnographic Characteristics in Patients with Prader–Willi Syndrome. Pediatr Pulmonol (2007) 42:881-7. doi: 10.1002/ppul.20673.

88. Wang P, Zhou W, Yuan W, Huang L, Zhao N, Chen X. Prader–Willi Syndrome in Neonates: Twenty Cases and Review of the Literature in Southern China. BMC Pediatr (2016) 16:1-6 doi: 10.1186/s12887-016-0662-2.

89. Irizarry KA, Miller M, Freemark M, Haqq AM. Prader Willi Syndrome: Genetics, Metabolomics, Hormonal Function, and New Approaches to Therapy. Adv Pediatr (2016) 63:47. doi: 10.1016/j.yapd.2016.04.005.

90. Perkins JA, Sie KC, Milczuk H, Richardson MA. Airway Management in Children with Craniofacial Anomalies. Cleft Palate- Craniofac J (1997) 34:135-40. doi: 10.1597/1545-1569_1997_034_0135_amicwc_2.3.co_2.

91. da Fontoura CS, Miller SF, Wehby GL, Amendt BA, Holton NE, Southard TE, et al. Candidate Gene Analyses of Skeletal Variation in Malocclusion. J Dent Res (2015) 94:913-20. doi: 10.1177/0022034515581643.

92. Zhang Y, Blackwell EL, McKnight MT, Knutsen GR, Vu WT, Ruest LB. Specific Inactivation of Twist1 in the Mandibular Arch Neural Crest Cells Affects the Development of the Ramus and Reveals Interactions with Hand2. Dev Dyn (2012) 241:924-40. doi: 10.1002/dvdy.23776.

93. Choi T, Lijten O, Mathijssen I, Wolvius E, Ongkosuwito E. Craniofacial Morphology and Growth in Muenke Syndrome, Saethre-Chotzen Syndrome, and Tcf12-Related Craniosynostosis. Clin Oral Investig (2022) 26:2927-36. doi: 10.1007/s00784-021-04275-y.

94. Akre H, Øverland B, Åsten P, Skogedal N, Heimdal K. Obstructive Sleep Apnea in Treacher Collins Syndrome. Eur Arch Otorhinolaryngol (2012) 269:331-7. doi: 10.1007/s00405-011-1649-0.

95. Plomp RG, Bredero-Boelhouwer HH, Joosten KF, Wolvius EB, Hoeve HL, Poublon RM, et al. Obstructive Sleep Apnoea in Treacher Collins Syndrome: Prevalence, Severity and Cause. Int J Oral Maxillofac Surg (2012) 41:696-701. doi: 10.1016/j.ijom.2012.01.018.

96. Ma X, Forte AJ, Persing JA, Alonso N, Berlin NL, Steinbacher DM. Reduced Three-Dimensional Airway Volume Is a Function of Skeletal Dysmorphology in Treacher Collins Syndrome. Plast Reconstr Surg (2015) 135:382e-92e. doi: 10.1097/PRS.0000000000000993.

97. Martelli-Junior H, Coletta RD, Miranda R-T, Barros L, Swerts M-S, Bonan P-R. Orofacial Features of Treacher Collins Syndrome. Med Oral Patol Oral Cir Bucal (2009) 14:344-8.

98. Sakai D, Trainor PA. Treacher Collins Syndrome: Unmasking the Role of Tcof1/Treacle. Int J Biochem Cell Biol. (2009) 41:1229-32. doi: 10.1016/j.biocel.2008.10.026.
